# Outbreak of SARS-CoV-2 B.1.617.2 (Delta) Variant Infections Among Incarcerated Persons in a Federal Prison — Texas, July–August 2021

**DOI:** 10.15585/mmwr.mm7038e3

**Published:** 2021-09-24

**Authors:** Liesl M. Hagan, David W. McCormick, Christine Lee, Sadia Sleweon, Lavinia Nicolae, Thomas Dixon, Robert Banta, Isaac Ogle, Cristen Young, Charles Dusseau, Shawn Salmonson, Charles Ogden, Eric Godwin, TeCora Ballom, Tara Ross, Hannah Browne, Jennifer L. Harcourt, Azaibi Tamin, Natalie J. Thornburg, Hannah L. Kirking, Phillip P. Salvatore, Jacqueline E. Tate

**Affiliations:** ^1^CDC COVID-19 Response Team; ^2^Epidemic Intelligence Service, CDC; ^3^Bureau of Prisons, U.S. Department of Justice.

Incarcerated populations have experienced disproportionately higher rates of COVID-19–related illness and death compared with the general U.S. population, due in part to congregate living environments that can facilitate rapid transmission of SARS-CoV-2, the virus that causes COVID-19, and the high prevalence of underlying medical conditions associated with severe COVID-19 (*1*,*2*). The SARS-CoV-2 B.1.617.2 (Delta) variant has caused outbreaks among vaccinated and unvaccinated persons in congregate settings and large public gatherings (*3*,*4*). During July 2021, a COVID-19 outbreak involving the Delta variant was identified in a federal prison in Texas, infecting 172 of 233 (74%) incarcerated persons in two housing units. The Federal Bureau of Prisons (BOP) partnered with CDC to investigate. CDC analyzed data on infection status, symptom onset date, hospitalizations, and deaths among incarcerated persons. The attack rate was higher among unvaccinated versus fully vaccinated persons (39 of 42, 93% versus 129 of 185, 70%; p = 0.002).^†^ Four persons were hospitalized, three of whom were unvaccinated, and one person died, who was unvaccinated. Among a subset of 70 persons consenting to an embedded serial swabbing protocol, the median interval between symptom onset and last positive reverse transcription–polymerase chain reaction (RT-PCR) test result in fully vaccinated versus unvaccinated persons was similar (9 versus 11 days, p = 0.37). One or more specimens were culture-positive from five of 12 (42%) unvaccinated and 14 of 37 (38%) fully vaccinated persons for whom viral culture was attempted. In settings where physical distancing is challenging, including correctional and detention facilities, vaccination and implementation of multicomponent prevention strategies (e.g., testing, medical isolation, quarantine, and masking) are critical to limiting SARS-CoV-2 transmission (*5*).

## Investigation and Response

On July 12, 2021, 18 persons incarcerated in a federal prison in Texas reported COVID-19–like symptoms to BOP health services staff members. All 18 received positive SARS-CoV-2 test results using the Abbott BinaxNOW COVID-19 Ag Card (rapid antigen) test; 11 were fully vaccinated. Three of these persons had reported to the on-site clinic on July 8 with symptoms including coryza, cough, headache, myalgia, or rhinorrhea but did not receive SARS-CoV-2 testing at that time.^§^ The 18 persons with positive test results lived in two interconnected units (unit A and unit B) that operated as a single cohort and housed 233 persons in 2- to 10-person cells without doors. Standard COVID-19 prevention protocols that were in place among incarcerated persons included mandatory masking in common areas, cohorting of housing units for daily activities, and head-to-toe sleeping arrangements. Among staff members, prevention protocols included mandatory masking and mandatory daily COVID-19 symptom screening and temperature checks (*5*).^¶^ Before the outbreak, incarcerated persons moved freely between units A and B and were together for meals, recreation, and work; they did not have contact with incarcerated persons housed in other units. After initial identification of COVID-19 cases, unit A was designated as a quarantine unit for persons with negative test results, and unit B was designated as a medical isolation unit for COVID-19 patients. Staff members assigned to units A and B rotated between these two units and to other units on the basis of daily staffing needs. 

During July 12–August 14, 2021, BOP staff members offered same-day SARS-CoV-2 rapid antigen testing to all 233 persons in units A and B reporting symptoms or known exposures; the entire quarantined cohort received testing from BOP during July 12–13 and again on July 14, July 19, July 22, August 2, and August 10 with a combination of rapid antigen and RT-PCR tests.** SARS-CoV-2 testing among staff members was voluntary and was performed off-site by staff members’ health care providers. A subset of 70 incarcerated persons in units A and B consented to a secondary investigation for which they reported symptom data through a questionnaire and provided nasal midturbinate swabs daily for up to 20 days after symptom onset. Specimens were tested by RT-PCR.^††^ Viral culture was attempted for RT-PCR–positive specimens from a nonrandom subset of participants.^§§^ Genomic sequencing was attempted for one RT-PCR–positive specimen from each participant, when possible.

COVID-19 vaccination was voluntary for BOP staff and incarcerated persons. In 2020, BOP worked with CDC to develop a vaccine prioritization plan in which all staff members were offered vaccination first, followed by incarcerated persons. Among incarcerated persons, those aged ≥65 years and those with underlying medical conditions associated with severe COVID-19 were the first to receive a COVID-19 vaccine. In this prison, the Pfizer-BioNTech vaccine was the first available, with first doses administered to incarcerated persons in January 2021.^¶¶^ Staff vaccination coverage in this report includes only doses administered as part of the BOP occupational health program. BOP was unable to determine the number of staff members who were vaccinated through other providers.

Information on vaccination, demographic characteristics, and underlying medical conditions was extracted from BOP electronic medical records for all 233 persons living in units A and B. Demographic characteristics, underlying medical conditions, and COVID-19–associated hospitalizations and deaths were compared by vaccination status and, among vaccinated persons, by vaccine product received. Attack rates were compared by demographic and medical characteristics, vaccination status and vaccine product, and time since vaccination. Descriptive statistics were calculated. Differences between groups were assessed using chi-square or Fisher’s exact tests. P-values <0.05 were considered statistically significant, adjusted for multiple comparisons using the Bonferroni correction method. Statistical analyses were performed using SAS (version 9.4; SAS Institute). This activity was reviewed and approved by the BOP Research Review Board and CDC and conducted consistent with applicable federal law and CDC policy.***

Among 233 incarcerated persons, 185 (79%) of whom were fully vaccinated, 172 (74%) received positive SARS-CoV-2 test results during July 12–August 14 (Supplementary Figure, https://stacks.cdc.gov/view/cdc/109901). Among a subset of 70 symptomatic persons providing swabs for serial testing, no significant difference was found in the median interval between reported symptom onset and last positive RT-PCR result in vaccinated versus unvaccinated persons (9 versus 11 days, respectively; p = 0.37) (Figure). Virus was cultured from one or more specimens from five of 12 (42%) unvaccinated and 14 of 37 (38%) fully vaccinated persons for whom viral culture was attempted. Genomic sequencing confirmed the AY.3 sublineage of the Delta variant in 58 specimens from 58 persons.

**FIGURE:**
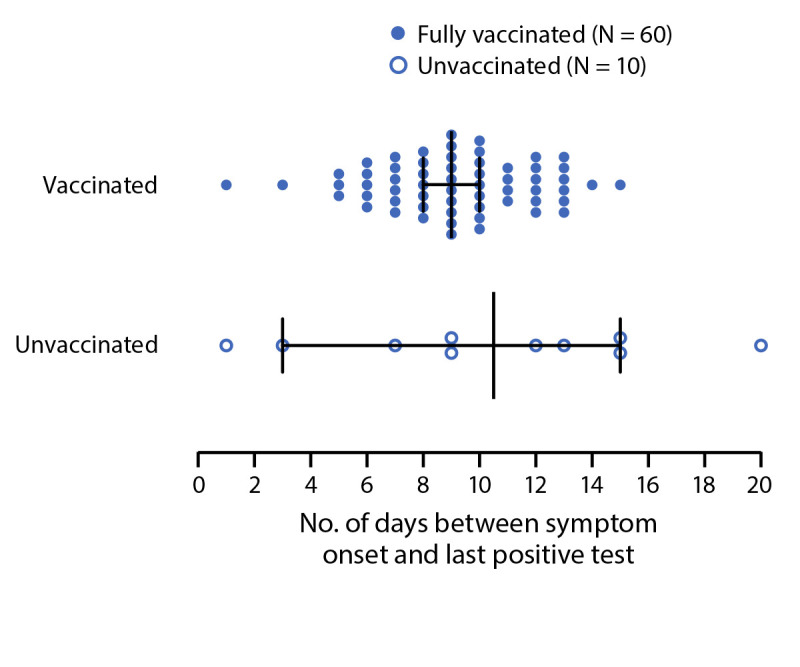
Number of days* between COVID-19 symptom onset and last positive SARS-CoV-2 reverse transcription–polymerase chain reaction test result among incarcerated persons^†^ in a federal prison, by vaccination status^§^ — Texas, July 19–August 9, 2021 **Abbreviation:** FDA = Food and Drug Administration. * Vertical lines indicate median number of days; horizontal lines indicate interquartile ranges. ^†^ A subset of 70 persons who consented to an embedded serial swabbing protocol. ^§^ Persons were considered fully vaccinated if ≥14 days had elapsed since they completed all recommended doses of an FDA-authorized COVID-19 vaccine series before symptom onset or date of first positive test.

Vaccination coverage was 79% among incarcerated persons in units A and B. Among fully vaccinated persons, 93 of 122 (76%) Pfizer-BioNTech recipients and 0 of 50 (0%) Moderna recipients had been vaccinated ≥4 months before the outbreak (p<0.001). A larger proportion of Pfizer-BioNTech recipients had diabetes (p = 0.02) or hypertension (p<0.001) than Moderna or Janssen COVID-19 vaccine recipients, and a higher proportion of Pfizer-BioNTech and Janssen recipients had a history of smoking (p<0.001) than Moderna recipients (Table 1).

**TABLE 1:** Vaccination status* among incarcerated persons in a federal prison, by demographic characteristics, underlying conditions, and COVID-19–associated hospitalizations and deaths — Texas, July 12–August 14, 2021

Characteristic	No. (%)			
	Total	Unvaccinated	Fully vaccinated	p-value^†^
**Total**	**233 (100)**	**42 (18)**	**185 (79)**	**—**
**Sex**				
Male	**233 (100)**	42 (18)	185 (79)	—
**Age group, yrs**	—	—	—	0.17
18–29 (Ref.)	**10 (4)**	3 (33)	6 (67)	Ref.
30–39	**63 (27)**	16 (26)	46 (74)	0.69
40–49	**68 (29)**	11 (17)	53 (83)	0.36
50–59	**65 (28)**	10 (15)	55 (85)	0.19
≥60	**27 (12)**	2 (7)	25 (93)	0.09
**Race/Ethnicity**	—	—	—	0.02
American Indian/Alaska Native	**5 (2)**	0 (—)	5 (100)	1.0
Asian	**3 (1)**	0 (—)	2 (100)	1.0
Black, non-Hispanic	**47 (20)**	16 (36)	29 (64)	<0.001^§^
Hispanic	**34 (15)**	7 (22)	25 (78)	0.22
White, non-Hispanic	**144 (62)**	19 (13)	124 (87)	Ref.
**Country of birth**				
Outside the United States	**10 (4)**	3 (33)	6 (67)	0.37
United States	**223 (96)**	39 (18)	179 (82)	
**Vaccination status**				
Fully vaccinated	**185 (79)**	—	185 (100)	—
Partially vaccinated	**6 (3)**	—	—	
Unvaccinated	**42 (18)**	42 (100)	—	
**Vaccine product received (among fully vaccinated)**				
Janssen (Johnson & Johnson)	**—**	—	13 (100)	—
Moderna	**—**	—	50 (100)	
Pfizer-BioNTech	**—**	—	122 (100)	
**Time from full vaccination to outbreak (among fully vaccinated)**				
≥2 wks to 2 mos	**—**	—	31 (100)	—
2–4 mos	**—**	—	61 (100)	
4–6 mos	**—**	—	93 (100)	
**Documented previous SARS-CoV-2 infection**				
No	**204 (88)**	35 (18)	164 (82)	0.34
Yes	**29 (12)**	7 (25)	21 (75)	
**Housing unit before outbreak**				
A	**146 (63)**	25 (18)	116 (82)	0.70
B	**87 (37)**	17 (20)	69 (80)	
**Underlying medical conditions^¶^**				
History of smoking**	**121 (52)**	14 (12)	105 (88)	0.006^§^
Overweight**^††^**	**89 (38)**	22 (25)	66 (75)	0.07
Obesity**^††^**	**101 (43)**	13 (13)	84 (87)	
Severe obesity**^††^**	**19 (8)**	1 (6)	17 (94)	
Hypertension	**90 (39)**	13 (15)	75 (85)	0.25
Diabetes	**29 (12)**	2 (7)	27 (93)	0.12
Moderate to severe asthma	**25 (11)**	3 (12)	21 (88)	0.58
Chronic obstructive pulmonary disease	**16 (7)**	1 (7)	14 (93)	0.32
Immunocompromised state	**4 (2)**	0 (—)	4 (100)	1.0
Chronic kidney disease	**3 (1)**	0 (—)	3 (100)	1.0
Cancer	**2 (1)**	0 (—)	2 (100)	1.0
Liver disease	**2 (1)**	1 (50)	1 (50)	0.34
Serious cardiac condition	**1 (0)**	1 (0)	0 (—)	0.19
HIV infection	**1 (0)**	0 (—)	1 (100)	1.0
**COVID-19 outcomes**				
Hospitalization	**4 (2)**	3 (75)	1 (25)	0.04^§^
Death	**1 (0)**	1 (100)	0 (—)	0.23

**Abbreviations:** BMI = body mass index; FDA = Food and Drug Administration; Ref. = referent group.

* Descriptive statistics were not calculated for partially vaccinated persons. Partially vaccinated persons were excluded from statistical comparisons by vaccination status. Persons were considered fully vaccinated if ≥14 days had elapsed since they completed all recommended doses of an FDA-authorized COVID-19 vaccine series before symptom onset or date of first positive test. Persons were considered partially vaccinated if they had not completed all doses of an FDA-authorized COVID-19 vaccine series or if they had received the final vaccine dose <14 days before symptom onset or date of first positive test.

^†^ P-values from chi-square test (when all cell sizes ≥5) or Fisher’s exact test (when any cell size <5).

^§^ Statistically significant difference; p-values <0.05 were considered statistically significant, adjusted for multiple comparisons using the Bonferroni correction method.

^¶^ No persons had pulmonary fibrosis or history of solid organ or stem cell transplant.

** Information on the type of product smoked was not available.

^††^ Overweight: BMI >25 kg/m^2^ but <30 kg/m^2^; obesity: BMI ≥30 kg/m^2^ but <40 kg/m^2^; severe obesity: BMI ≥40 kg/m^2^.

Attack rates were higher among unvaccinated persons (39 of 42; 93%) than among fully vaccinated persons (129 of 185; 70%) (p = 0. 002) and among persons vaccinated ≥4 months before the outbreak (83 of 93; 89%) than among those vaccinated 2 weeks to 2 months before the outbreak (19 of 31; 61%) (p<0.001) (Table 2).

**TABLE 2:** SARS-CoV-2 attack rates among incarcerated persons in a federal prison, by demographic characteristics, vaccination status, COVID-19 vaccine product, and underlying conditions — Texas, July 12–August 14, 2021

Characteristic	Total (column %)	No. of cases	Attack rate, %	p-value*
**Total **	**233 (100)**	**172**	**74**	**—**
**Vaccination status^†^**	—	—	—	0.003^§^
Unvaccinated	**42 (18)**	39	93	0.002^§^
Partially vaccinated	**6 (3)**	4	67	1.0
Fully vaccinated	**185 (79)**	129	70	Ref.
**Vaccine product (among fully vaccinated)**	—	—	—	<0.001^§^
Janssen (Johnson & Johnson)	**13 (7)**	10	77	0.03
Moderna	**50 (27)**	20	40	Ref.
Pfizer-BioNTech	**122 (66)**	99	81	<0.001^§^
**Time from full vaccination to outbreak (among fully vaccinated)**				<0.001^§^
≥2 wks to 2 mos	**31 (17)**	19	61	Ref.
2–4 mos	**61 (33)**	27	44	0.12
4–6 mos	**93 (50)**	83	89	<0.001^§^
**Sex**				
Male	**233 (100)**	172	74	—
**Age group, yrs**	—	—	—	0.46
18–29	**10 (4)**	6	60	Ref.
30–39	**63 (27)**	43	68	0.72
40–49	**68 (29)**	50	74	0.46
50–59	**65 (28)**	52	80	0.22
≥60	**27 (12)**	21	78	0.41
**Race/Ethnicity**	—	—	—	0.16
American Indian/Alaska Native	**5 (2)**	3	60	0.31
Asian	**3 (1)**	3	100	1.0
Black, non-Hispanic	**47 (20)**	31	66	0.08
Hispanic	**34 (15)**	22	65	0.09
White, non-Hispanic	**144 (62)**	113	78	Ref.
**Country of birth**				
Outside United States	**10 (4)**	9	90	0.46
United States	**223 (96)**	163	73	
**Housing unit before outbreak**				
Unit A	**146 (63)**	107	73	0.81
Unit B	**87 (37)**	65	75	
**Underlying medical conditions**				
History of smoking^¶^	**121 (52)**	88	73	0.69
Hypertension	**90 (39)**	73	81	0.05
Overweight**	**89 (38)**	64	72	0.55
Obesity**	**101 (43)**	76	75	
Severe obesity**	**19 (8)**	16	84	
Moderate to severe asthma	**25 (11)**	21	84	0.34
Diabetes	**29 (12)**	26	90	0.04^§^
Chronic obstructive pulmonary disease	**16 (7)**	15	94	0.08
Chronic kidney disease	**3 (1)**	3	100	0.57
Immunocompromised state	**4 (2)**	3	75	1.0
Liver disease	**2 (1)**	2	100	1.0
Cancer	**2 (1)**	1	50	0.46
Serious cardiac condition	**1 (0.4)**	1	100	1.0
HIV infection	**1 (0.4)**	1	100	1.0

**Abbreviations:** BMI = body mass index; FDA = Food and Drug Administration; Ref. = referent group.

* P-values from chi-square test (when all cell sizes ≥5) or Fisher’s exact test (when any cell size <5).

^†^ Persons were considered fully vaccinated if ≥14 days had elapsed since they completed all recommended doses of an FDA-authorized COVID-19 vaccine series before symptom onset or date of first positive test. Persons were considered partially vaccinated if they had not completed all doses of an FDA-authorized COVID-19 vaccine series or if they had received the final vaccine dose <14 days before symptom onset or date of first positive test.

^§^ Statistically significant difference; p-values <0.05 were considered statistically significant, adjusted for multiple comparisons using the Bonferroni correction method.

^¶^ Information on type of product smoked was not available.

** Overweight: BMI >25 kg/m^2^ but <30 kg/m^2^; obesity: BMI ≥30 kg/m^2^ but <40 kg/m^2^; severe obesity: BMI ≥40 kg/m^2^.

Among both persons with and without a previous SARS-CoV-2 infection, the attack rate was lower among fully vaccinated versus unvaccinated persons (1 of 21 [5%] versus 4 of 7 [57%], p = 0.008; 128 of 164 [78%] versus 35 of 35 [100%], p<0.001) (Supplementary Table, https://stacks.cdc.gov/view/cdc/109901). Among fully vaccinated persons without a previous SARS-CoV-2 infection, the attack rate was higher among Pfizer-BioNTech recipients (99 of 117; 85%) than among Moderna recipients (19 of 35; 54%) (p<0.001).

Among 172 infected persons, four (2%) were hospitalized for COVID-19, including three (8%) of 39 unvaccinated patients, and one (1%) of 129 fully vaccinated patients (p = 0.04). One (3%) of the unvaccinated hospitalized patients required endotracheal intubation and mechanical ventilation and died in the hospital (Table 1).^†††^

Nine of 275 (3%) staff members, four of whom worked in units A or B, reported a positive SARS-CoV-2 test result during the outbreak and were restricted from work per BOP policy. BOP administered COVID-19 vaccine to 37% of staff members in the prison.

## Discussion

This study demonstrates the potential for SARS-CoV-2 Delta variant outbreaks in congregate settings including correctional and detention facilities, even among resident populations with high vaccination coverage. In this outbreak involving almost three fourths of the incarcerated population in the affected housing units, fewer hospitalizations and deaths occurred among vaccinated than unvaccinated persons, highlighting vaccination as an important strategy to reduce serious COVID-19–related illness and death in congregate settings. In addition, the high number of infections in vaccinated persons, comparable duration of positive RT-PCR test results after symptom onset regardless of vaccination status, and presence of infectious virus in specimens from both unvaccinated and vaccinated infected persons underscore the importance of implementing and maintaining multiple COVID-19 prevention strategies in settings where physical distancing is challenging, even when vaccination coverage is high. Prevention strategies that were in place during this outbreak, including promptly separating infected and exposed persons and cohorting housing units for daily activities, might have prevented the outbreak from spreading to other areas of the prison.

Three of the four hospitalizations and the only death occurred in unvaccinated persons. These findings are consistent with a previous study in which vaccination with a COVID-19 mRNA vaccine (Pfizer-BioNTech or Moderna) reduced the risk for hospitalization associated with Delta variant infection (*6*). These findings reinforce the critical importance of vaccination in reducing risk for severe illness and death from SARS-CoV-2 Delta variant infections, particularly in congregate settings.

Natural infection with SARS-CoV-2 confers some degree of immunity, although the duration of protection is unknown (*7*). In this outbreak, the lowest attack rate occurred among fully vaccinated persons with previous infection, highlighting the importance of vaccination, even among persons with previous infection. In addition, attack rates in persons without previous infection were higher among Pfizer-BioNTech recipients than among Moderna recipients. In a recent study, the Moderna vaccine was found to be more effective at preventing COVID-19–related hospitalizations among U.S. adults without immunocompromising conditions (*6*). In this outbreak, attack rates were also higher in persons who were vaccinated ≥4 months before the outbreak compared with persons vaccinated more recently. Because all persons vaccinated ≥4 months before the outbreak received the Pfizer-BioNTech vaccine, determining the independent impact of vaccine product versus time since vaccination was not possible. Additional research is warranted to assess the duration of vaccine-induced and natural immunity, as well as the duration of infectious virus shedding by vaccinated and unvaccinated infected persons.

BOP records indicate that nearly two thirds of staff members in this prison were unvaccinated, and at least nine were infected during this outbreak. In addition, during the 2 weeks before the outbreak, community transmission was high.^§§§^ SARS-CoV-2 can be introduced into correctional facility populations and back into the community through daily entry and exit of staff members and interfacility transfers of incarcerated persons, and the identification of a single viral lineage among all sequenced specimens in this outbreak suggests a single introduction of the virus into the prison (*8*). Bidirectional connections between correctional facilities and communities highlight the importance of high vaccination coverage among both staff members and incarcerated persons, early diagnostic testing, routine screening testing when community transmission is high, maintaining consistent assignments of staff members for each housing unit, and excluding staff members from work when they are symptomatic or have COVID-19 (*5*,*9*).

The findings in this report are subject to at least five limitations. First, although rapid antigen testing can identify cases quickly, its limited sensitivity for detecting infections in asymptomatic patients can underestimate attack rates (*10*). Second, transmission might have preceded initial identification of cases, resulting in an underestimation of total cases. Third, it is uncertain whether lower attack rates by vaccine product were caused by differences in waning vaccine-induced immunity, varying levels of protection among vaccine products, or differences in exposure level among persons who received different vaccine products. Fourth, testing was not mandatory for BOP staff members, limiting the ability to confirm the total numbers of COVID-19 cases. Finally, RT-PCR–positive specimens were not selected randomly for viral culture and thus are not representative of all vaccinated and unvaccinated participants.

During a COVID-19 outbreak in a federal prison involving the highly transmissible SARS-CoV-2 Delta variant, transmission was high among vaccinated and unvaccinated persons. Although hospitalizations, deaths, and attack rates were higher among unvaccinated than vaccinated persons, the duration of positive serial test results was similar between these two groups, and infectious virus was cultured from both vaccinated and unvaccinated participants. Widespread vaccination among incarcerated persons and staff members in coordination with other prevention strategies, including early diagnostic testing for all persons with any COVID-19–like symptoms, screening testing, medical isolation, quarantine, masking, and physical distancing where possible, remain critical to limiting SARS-CoV-2 transmission and COVID-19–related illness and death in congregate settings, including correctional and detention facilities (*5*).

**Summary**

**What is already known about this topic?**

Incarcerated populations have experienced disproportionately higher rates of COVID-19–related illness and death.

**What is added by this report?**

During a COVID-19 outbreak involving the Delta variant in a highly vaccinated incarcerated population, transmission rates were high, even among vaccinated persons. Although attack rates, hospitalizations, and deaths were higher among unvaccinated than among vaccinated persons, duration of positive serial test results was similar for both groups. Infectious virus was cultured from vaccinated and unvaccinated infected persons.

**What are the implications for public health practice?**

Even with high vaccination rates, maintaining multicomponent prevention strategies (e.g., testing and masking for all persons and prompt medical isolation and quarantine for incarcerated persons) remains critical to limiting SARS-CoV-2 transmission in congregate settings where physical distancing is challenging.

## Footnotes

* These authors contributed equally to the report.

† All persons included in the vaccine coverage calculation categorized as vaccinated were fully vaccinated. Persons were considered fully vaccinated if ≥14 days had elapsed since they completed all recommended doses of a Food and Drug Administration (FDA)-authorized COVID-19 vaccine series before symptom onset or date of first positive test. Persons were considered partially vaccinated if they had not completed all doses of an FDA-authorized COVID-19 vaccine series or if they had received the final vaccine dose <14 days before symptom onset or date of first positive test. Partially vaccinated persons were excluded from statistical comparisons by vaccination status.

§ These persons were identified by a review of on-site clinic records. Clinic records and discussions with on-site staff members suggested that clinicians thought symptoms were likely due to other causes, given a lack of known cases in the prison since January 2021.

¶ Alcohol-based hand sanitizer was provided in staff-only areas. Mitigation measures among incarcerated persons beyond mandatory masking in common areas included on-site voluntary vaccination provided by BOP; prompt medical isolation of persons testing positive for SARS-CoV-2 and quarantine of exposed persons testing negative; consistent cohorting of housing units for daily activities including meals, recreation, and work assignments; and head-to-toe sleeping arrangements. Signs encouraging frequent hand hygiene were posted throughout the prison, and soap was provided without cost to incarcerated persons. Environmental mitigation measures included regular disinfection of common areas and high-touch surfaces and provision of individual bottles of disinfectant to incarcerated persons for use in their personal spaces. Hard plastic barriers were installed in visitation areas to prevent physical contact between incarcerated persons and visitors.

** Rapid antigen testing was used during the early and middle phases of the outbreak to identify cases quickly and facilitate timely separation of infected persons from those with negative test results. RT-PCR testing was used in the late phase of the outbreak to confirm no new cases had occurred before lifting quarantine precautions.

††
https://www.fda.gov/media/139743/download

§§ RT-PCR–positive specimens were chosen for viral culture to include both vaccinated and unvaccinated participants and to represent different points in time since first positive diagnostic test. All specimens chosen for culture from vaccinated and unvaccinated participants had a cycle threshold value of <38 and were collected from 3 days before through 13 days after symptom onset.

¶¶
https://www.sciencedirect.com/science/article/pii/S0264410X21010781?via%3Dihub

*** 45 C.F.R. part 46, 21 C.F.R. part 56; 42 U.S.C. Sect. 241(d); 5 U.S.C. Sect. 552a; 44 U.S.C. Sect. 3501 et seq.

††† The unvaccinated hospitalized patient who died was aged 50–59 years and had obesity, hypertension, and a history of smoking. Among the remaining two hospitalized unvaccinated patients, one was aged 40–49 years and had obesity, and the other was aged 40–49 years and had overweight and moderate to severe asthma. The vaccinated hospitalized patient was aged 50–59 years and had obesity, type II diabetes, hypertension, and a history of smoking.

§§§ https://www.cdc.gov/coronavirus/2019-ncov/covid-data/covidview/index.html. CDC defines community transmission as high when ≥10.0% of nucleic acid amplification tests (NAATs) in the previous 7 days have been positive or when ≥100 new cases per 100,000 persons have occurred in the previous 7 days. In the 2 weeks before the outbreak described in this report, median NAAT test positivity was 17.8% (range = 5%–39.5%) in counties surrounding the affected federal prison.

## References

[1] Maruschak L, Bronson J, Alper M. Medical problems reported by prisoners, survey of prison inmates, 2016. Washington, DC: US Department of Justice, Bureau of Justice Statistics; 2021. https://bjs.ojp.gov/sites/g/files/xyckuh236/files/media/document/mprpspi16st.pdf

[2] Saloner B, Parish K, Ward JA, DiLaura G, Dolovich S. COVID-19 cases and deaths in federal and state prisons. JAMA 2020;324:602–3. 10.1001/jama.2020.12528 32639537 PMC7344796

[3] Nanduri S, Pilishvili T, Derado G, Effectiveness of Pfizer-BioNTech and Moderna vaccines in preventing SARS-CoV-2 infection among nursing home residents before and during widespread circulation of the SARS-CoV-2 B.1.617.2 (Delta) variant—National Healthcare Safety Network, March 1–August. MMWR Morb Mortal Wkly Rep 2021;70:1163–6. 10.15585/mmwr.mm7034e3 34437519 PMC8389386

[4] Brown CM, Vostok J, Johnson H, Outbreak of SARS-CoV-2 infections, including COVID-19 vaccine breakthrough infections, associated with large public gatherings—Barnstable County, Massachusetts, July 2021. MMWR Morb Mortal Wkly Rep 2021;70:1059–62. 10.15585/mmwr.mm7031e2 34351882 PMC8367314

[5] CDC. Interim guidance on management of coronavirus disease 2019 (COVID-19) in correctional and detention facilities. Atlanta, GA: US Department of Health and Human Services, CDC; 2021. Accessed August 20, 2021. https://www.cdc.gov/coronavirus/2019-ncov/community/correction-detention/guidance-correctional-detention.html

[6] Self WH, Tenforde MW, Rhoads JP, Comparative effectiveness of Moderna, Pfizer-BioNTech, and Janssen (Johnson & Johnson) vaccines in preventing COVID-19 hospitalizations among adults without immunocompromising conditions—United States, March–August 2021. MMWR Morb Mortal Wkly Rep 2021. Epub September 17, 2021. 10.15585/mmwr.mm7038e1 PMC8459899 34555004

[7] Hansen CH, Michlmayr D, Gubbels SM, Mølbak K, Ethelberg S. Assessment of protection against reinfection with SARS-CoV-2 among 4 million PCR-tested individuals in Denmark in 2020: a population-level observational study. Lancet 2021;397:1204–12. 10.1016/S0140-6736(21)00575-4 33743221 PMC7969130

[8] Wallace M, Hagan L, Curran KG, COVID-19 in correctional and detention facilities—United States, February–April 2020. MMWR Morb Mortal Wkly Rep 2020;69:587–90. 10.15585/mmwr.mm6919e1 32407300

[9] Lewis NM, Salmanson AP, Price A, Community-associated outbreak of COVID-19 in a correctional facility—Utah, September 2020–January 2021. MMWR Morb Mortal Wkly Rep 2021;70:467–72. 10.15585/mmwr.mm7013a2 33793464 PMC8022878

[10] Prince-Guerra JL, Almendares O, Nolen LD, Evaluation of Abbott BinaxNOW rapid antigen test for SARS-CoV-2 infection at two community-based testing sites—Pima County, Arizona, November 3–17, 2020. MMWR Morb Mortal Wkly Rep 2021;70:100–5. 10.15585/mmwr.mm7003e3 33476316 PMC7821766

